# SHAP-based explainable AI framework for autism severity classification using 3D motor biomarkers

**DOI:** 10.3389/fpsyt.2026.1751654

**Published:** 2026-03-23

**Authors:** Yelda Fırat

**Affiliations:** Department of Computer Engineering, Mudanya University, Bursa, Türkiye

**Keywords:** autism spectrum disorder, motor biomarkers, random forest, SHAP analysis, violence level classification

## Abstract

**Introduction:**

Early Autism spectrum disorder (ASD) diagnosis is critical for intervention, yet current methods rely on subjective clinical observations. This study develops objective tools to classify ASD severity using 3D motor movement analysis, addressing motor abnormalities as core diagnostic features.

**Methods:**

A Random Forest (RF) model classified three severity levels using 463 motor features from 25 Kinect V2 joint points. Data from 109 children (50 typical, 50 moderate ASD, 9 severe ASD) were validated via 5-fold cross-validation and two held-out test sets (20% each). Shapley Additive Explanations (SHAP) analysis identified critical motor biomarkers.

**Results:**

The model achieved 84.6±10.9% accuracy (5-fold cross-validation) and 86.4% accuracy (internal and held-out test sets). For severe ASD, the model achieved 100% classification accuracy on synthetic test data (4/4 cases; 95% CI: 39.8%-100.0%). However, this result represents a methodological proof-of-concept rather than clinical validation, as severe ASD features were synthetically generated from moderate ASD data and the model has not been validated on real Kinect-derived severe ASD motor data. SHAP analysis identified wrist movements, knee trajectories, and elbow-to-foot distances as key motor biomarkers for severity classification.

**Discussion:**

This Kinect-based approach with RF and SHAP offers effective, interpretable ASD severity assessment for typical and moderate ASD classes, with promising methodological foundations for severe ASD pending validation on real data.

## 1 Introduction

Autism spectrum disorder (ASD), affecting 1 in 36 U.S. children (1), is characterized by social communication difficulties, repetitive behaviors, and restricted interests. Current diagnostic methods rely on subjective clinical observation, often delaying diagnosis beyond age four and missing early intervention opportunities. Motor abnormalities—balance, gait, postural control, and stereotypical movements—are core ASD features. Motor impairments may serve as potential early diagnostic biomarkers (2, 3).

Machine learning and 3D motion analysis enable objective assessment of ASD. Microsoft Kinect captures motor characteristics in three dimensions at low cost. Machine learning achieves high accuracy in classifying ASD patterns. Random Forest (RF) offers performance and flexibility, while Shapley Additive Explanations (SHAP) facilitates clinical interpretation and increases professional confidence (4).

Balance, gait, and motor coordination impairments in children with ASD (3) serve as potential biomarkers. Gait analysis reveals timing variability (5) and differences in spatiotemporal parameters (6) compared to typical development. Machine learning combined with gait deviations enables effective early detection (7), demonstrating motor characteristics as objective diagnostic markers.

3D skeleton-based motion analysis is widely used in ASD detection. Kinect-based approaches include deep learning (8), walking analysis (9), stereotypical movement tracking (hand flapping, rocking, spinning) (10), and real-time action recognition via convolutional neural networks (11). The Kinect V2 dataset (12) provides resources for analyzing motor profiles in ASD and typical development.

Automatic stereotypical motor movement (SMM) detection is important in ASD diagnosis. Studies employed deep learning for video analysis (13), lower-body movement tracking (14), and wearable sensors (15), demonstrating that movement analysis provides objective diagnostic data.

Machine learning in ASD detection has increased rapidly. Studies show promise for gait analysis (16), high accuracy via dual-modal features (17), social interactions (18), and minimal medical data (19), and voice/behavioral data analysis (20), demonstrating effectiveness.

RF is widely used in ASD classification for behavioral aggression (21), cognitive profiling (22), and biomarker discovery across ages and intelligence levels (23), demonstrating high performance and interpretability.

Explainable AI (XAI) is increasingly important in ASD detection. Studies examined XAI applications (4), developed high-accuracy models (24), revealed subtype differences (25), and analyzed functional connectivity (26). SHAP effectively explains predictions and identifies important features.

Motor skills are important in ASD diagnosis. Motor and sensory characteristics improve classification with clinical scales (27), while atypical kinematics reveal control problems (28). Motor characteristics are fundamental for ASD diagnosis.

Motor analysis and machine learning assess ASD but focus on binary classification, leaving a research gap in severity-level classification and interpretability.

Building on these developments, several studies have demonstrated the potential for objective ASD classification through motor analysis using diverse sensor technologies. Early pioneering work by Crippa et al. (29) achieved 96.7% accuracy using Kinect-based upper limb kinematics with Support Machine Vectors (SVM), while Anzulewicz et al. (30) demonstrated 93% accuracy through tablet-based gesture analysis using ensemble methods. Li et al. (31) explored imitation-based classification using motion capture, achieving 73% accuracy with SVM and RF. More recently, Freud et al. (32) achieved over 84% accuracy in distinguishing autistic from non-autistic young adults using grasping kinematics captured with only two passive markers, demonstrating that minimal sensor configurations can provide robust classification performance. Similarly, Kojovic et al. (33) employed 2D video-based pose estimation combined with convolutional neural networks to achieve 80.4% accuracy in classifying young children with ASD. More advanced approaches include Su et al. (34), who achieved 78.1% accuracy using deep learning (Multilayer Perceptron -MLP) to classify reach-and-place movements captured with Inertial Measurement Unit (IMU) sensors, and Altozano et al. (35), who introduced 3DCNN ResNets for full-body kinematic assessment in virtual reality environments, achieving 85 ± 3% accuracy. These studies highlight the growing evidence base for motor-based biomarkers in ASD assessment across different age groups, sensor modalities, and motor tasks. A comprehensive summary of machine learning studies for ASD classification using motor features is presented in **Table 1**.

**Table 1:** Summary of machine learning studies for ASD classification using motor features.

Study	Year	Sensor	Sample	ML method	Accuracy	Key features
Crippa et al.	2015	Kinect	30	SVM	96.7%	Upper limb
Anzulewicz et al.	2016	Tablet	82	Ensemble	93%	Gestures
Li et al.	2017	Motion capture	28	SVM, RF	73%	Imitation
Kojovic et al.	2021	2D video	136	CNN	80.4%	Full-body pose
Simeoli et al.	2024	Review	Various	Various	70-97%	Motor patterns
Su et al.	2024	IMU	41	MLP	78.1%	Reach-place
Altozano et al.	2025	VR	Various	3DCNN	85 ± 3%	Full-body
Shin et al.	2025	Kinect v2	100	Dual-stream	95.4%	Skeleton
Freud et al.	2025	Motion tracking	60	Multiple	>84%	Grasping
Ganai et al.	2025	Gait	Various	SVM, RF	Various	Gait
Present study	2026	Kinect V2	109	RF	86.4%	3D skeleton, SHAP

As shown in **Table 1**, existing machine learning studies for ASD classification using motor features predominantly focus on binary classification (ASD vs. typical development), with limited attention to severity-level differentiation. The specific objectives of this study are: (1) to develop a RF-based classification model for distinguishing three ASD severity levels (typical development, moderate ASD, and severe ASD) using 3D motor features; (2) to identify and interpret key motor biomarkers contributing to severity classification through SHAP analysis; and (3) to evaluate the model’s performance and generalizability using multiple validation strategies including cross-validation and held-out test sets.

## 2 Methods

### 2.1 Dataset

This study utilized the “Three-dimensional dataset combining gait and full-body movement of children with autism spectrum disorders” collected using Kinect V2 and published on Zenodo (12). The dataset contains 3D joint coordinates and motor data from 109 children in a controlled laboratory environment. Participants: typically developing (n=50), moderate ASD (n=50), and severe ASD (n=9). Kinect V2 recorded 25 joint points (head, neck, shoulders, elbows, wrists, hands, spine, hips, knees, ankles, feet) in real time (X, Y, Z coordinates).

Dataset creators pre-calculated 463 motor features in Excel format: inter-joint Euclidean distances, range of motion (ROM) metrics, gait parameters (speed, step length, cycle time), and temporal characteristics. Augmented data (7 geometric transformations: rotation, mirroring, scaling) were available for the Typical and Moderate ASD groups; only original recordings were used.

For Severe ASD, the dataset contains 9 video recordings (Samsung Note 9) without Kinect V2 data. Creators could not set up Kinect V2 in controlled environments for severe cases, so they collected videos in clinical settings. Extracting 463 features from videos was technically impossible (missing 3D coordinates, depth information, and time series data). Therefore, synthetic features were created by averaging Moderate ASD vectors and adding Gaussian noise (μ=0, σ=0.15), preserving the 463-feature structure for consistent comparisons. Critically, this means the model was trained and evaluated on synthetically generated severe ASD data, not real Kinect-derived motor measurements. Consequently, the model has not been validated on real severe ASD Kinect data, and severe ASD classification performance should be interpreted as methodological rather than clinical.

Data split: training (n=65; 30 Typical, 30 Moderate, 5 Severe), internal test (n=22; 10 Typical, 10 Moderate, 2 Severe), held-out test (n=22; 10 Typical, 10 Moderate, 2 Severe). The held-out test set was reserved solely for final validation. Note that both test sets were drawn from the same original dataset using the same sensor, protocol, and feature extraction pipeline, representing reserved subsets from the same data distribution rather than independent external validation. Ethical approval and parental consent were obtained.

### 2.2 Data processing

**Figure 1:**
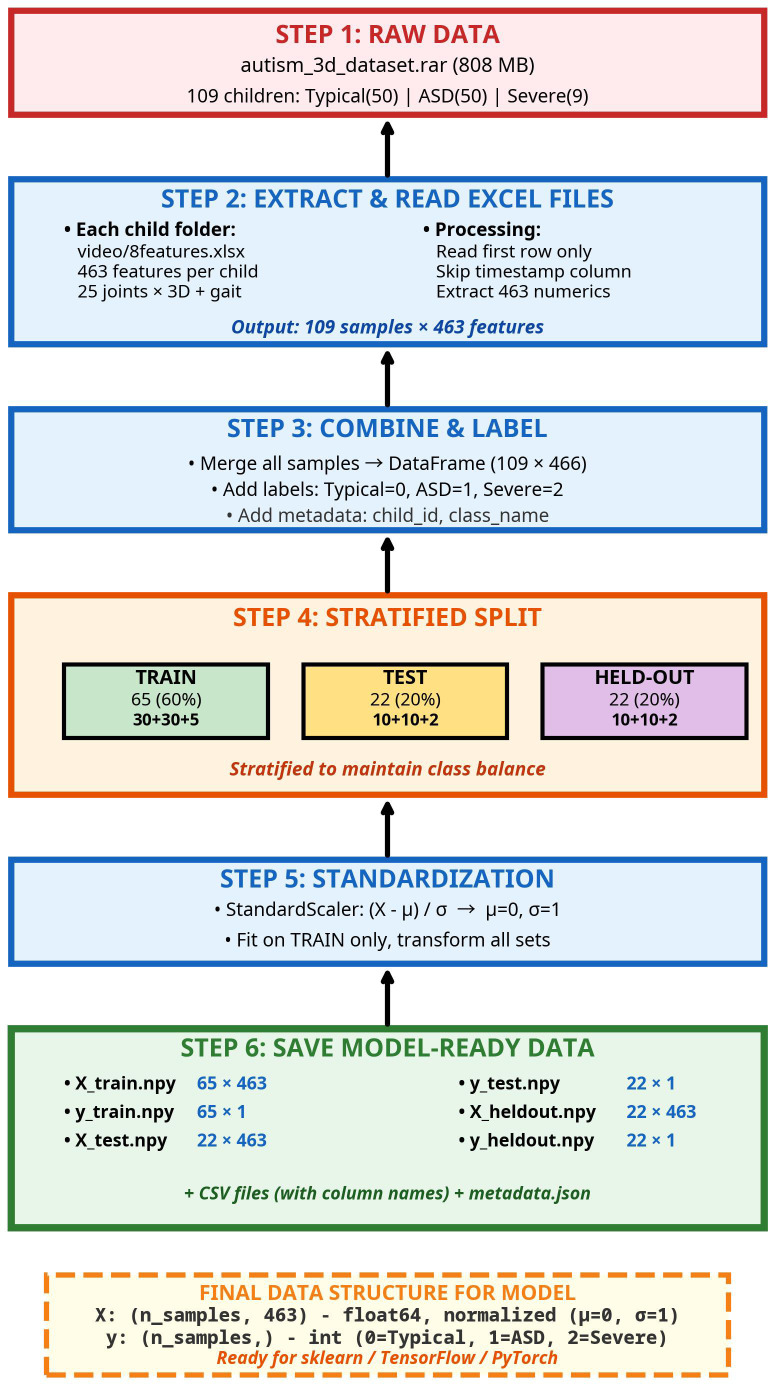
Data preparation flowchart.

The data preprocessing process has been designed as a systematic pipeline comprising stages that transform raw datasets into data ready for model training, as shown in **Figure 1**.

As shown in **Figure 1**, data preprocessing involved six stages. First, raw data from 109 children were extracted. Second, 463 video-level statistical features were extracted per child, omitting timestamps to obtain a single 463-dimensional vector. Third, data were combined into a dataframe (109 × 466) with class labels (Typical=0, Moderate ASD = 1, Severe ASD = 2). Fourth, stratified sampling divided data into training, internal test, and held-out test sets as described in Section 2.1. Fifth, Z-score normalization (μ=0, σ=1) was applied using training set parameters. Finally, data were saved in NumPy and CSV formats with feature names and metadata.

**Figure 2:**
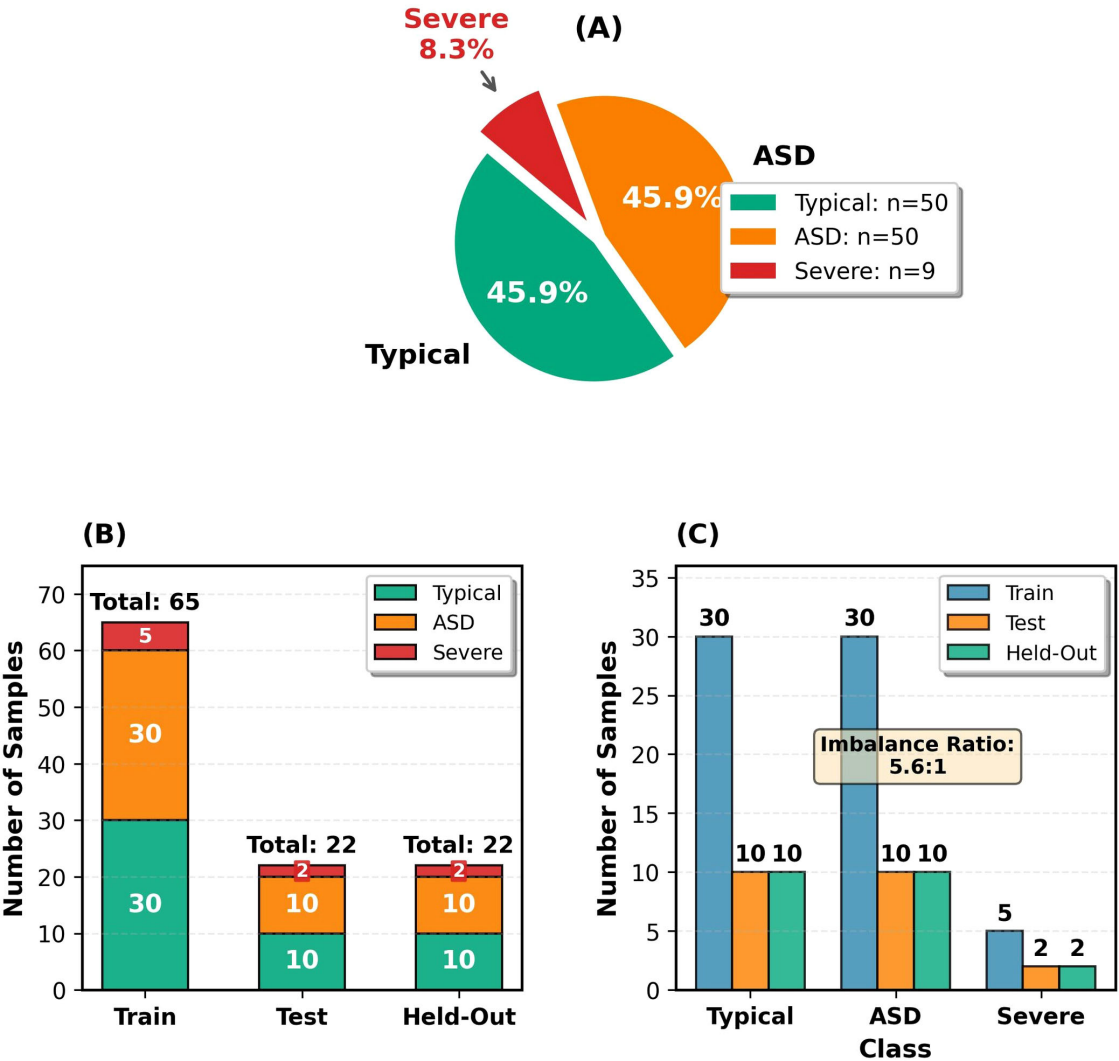
Data distribution and class balance analysis. **(A)** Overall class distribution. **(B)** Train/Test/Held-Out split. **(C)** Class balance analysis.

The class distribution of the data set and its division into subsets are shown in **Figure 2**.

The class distribution is shown in **Figure 2**. **Figure 2A** shows 45.9% Typical (n=50), 45.9% Moderate ASD (n=50), and 8.3% Severe ASD (n=9). **Figure 2B** displays the stratified sampling distribution across subsets. **Figure 2C** highlights class imbalance, particularly for Severe ASD. The held-out test set was reserved solely for final validation to evaluate real-world performance and minimize overfitting. No missing data were detected. All operations used Python 3.11.

### 2.3 Feature extraction

This study utilized 463 pre-calculated motor features from 25 Kinect V2 joint points (head, neck, shoulders, elbows, wrists, hands, fingertips, thumbs, spine levels, hips, knees, ankles, feet). Features are categorized into three types: (1) Raw joint coordinates (X, Y, Z axes for each joint); (2) Inter-joint Euclidean distances between all joint pairs, with systematic naming (e.g., ElLTFoL_X: left elbow to left foot X-axis distance; FoRTKeR_X: right foot to right knee X-axis distance); (3) ROM metrics expressing maximum-minimum position differences on X and Y axes (e.g., RomHaRx_X: right hand X-axis range of motion).

Features were extracted from walking and full-body motion recordings with summary statistics (mean, standard deviation, minimum, maximum). The naming system uses standard abbreviations: El (Elbow), Fo (Foot), Ke (Knee), Ha (Hand), Sh (Shoulder), ROM, L (Left), R (Right), T (To), ensuring anatomical clarity and interpretability.

**Figure 3:**
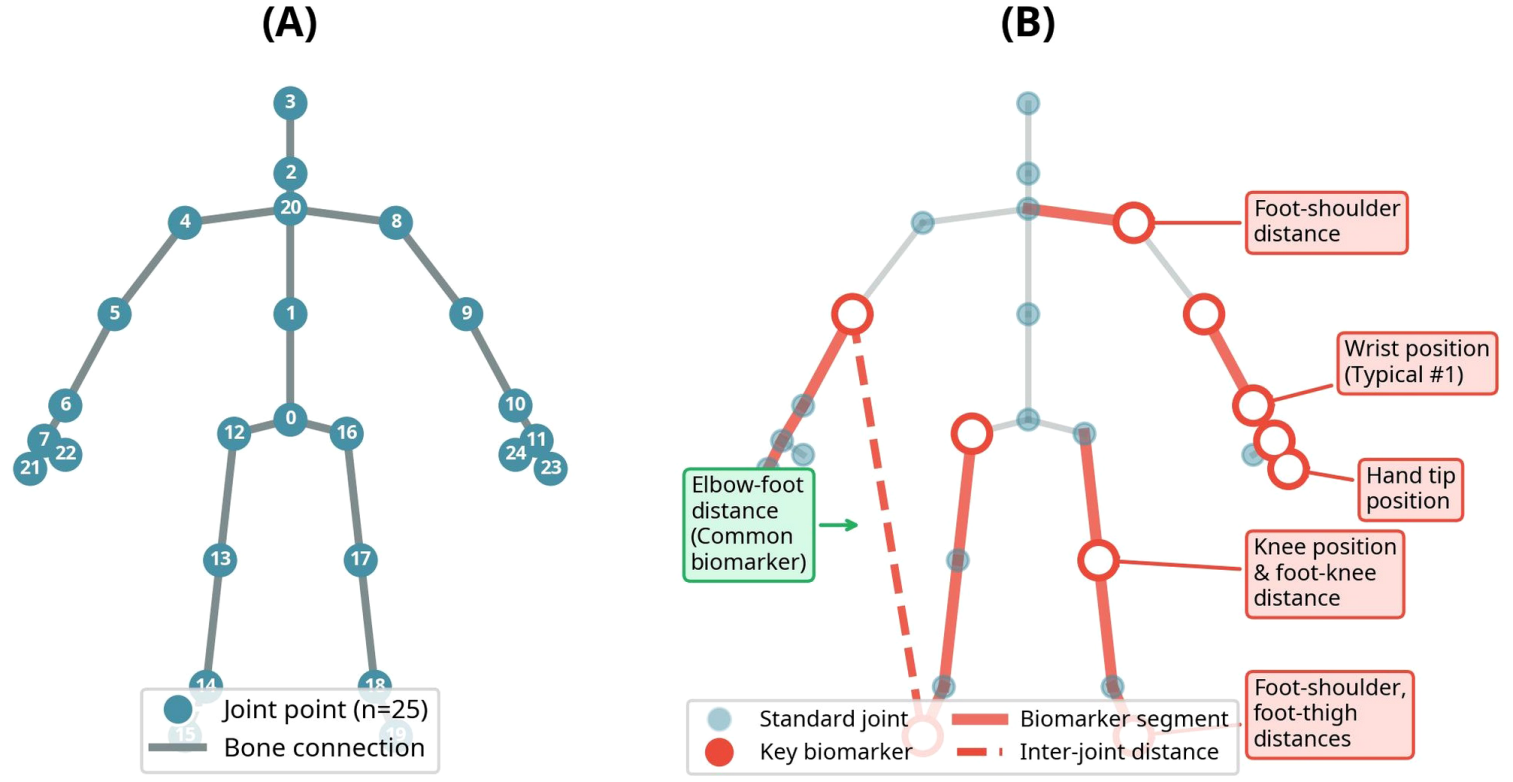
Kinect V2 skeleton structure and SHAP-identified motor biomarkers. **(A)** Kinect V2 skeleton with 25 joint points. **(B)** Key motor biomarkers identified by SHAP analysis.

All 463 features were used for the Typical and Moderate ASD groups. For Severe ASD, synthetic features were used as described in Section 2.1. No additional feature extraction or selection was performed; RF’s natural feature selection mechanism was employed. The Kinect V2 skeleton structure and SHAP-identified key motor biomarkers are illustrated in **Figure 3**.

As shown in **Figure 3**, the Kinect V2 sensor tracks 25 joint points representing the full-body skeletal structure. **Figure 3A** displays the complete skeleton with indexed joint points (0-24), while **Figure 3B** illustrates the anatomical locations of key motor features used in this study, including wrist position, knee position, and inter-joint distance measurements such as elbow-foot distance (ElLTFoL_X). The identification of which specific features emerged as most discriminative biomarkers is presented in Section 3.4 (SHAP Analysis Results).

### 2.4 Modelling

**Figure 4:**
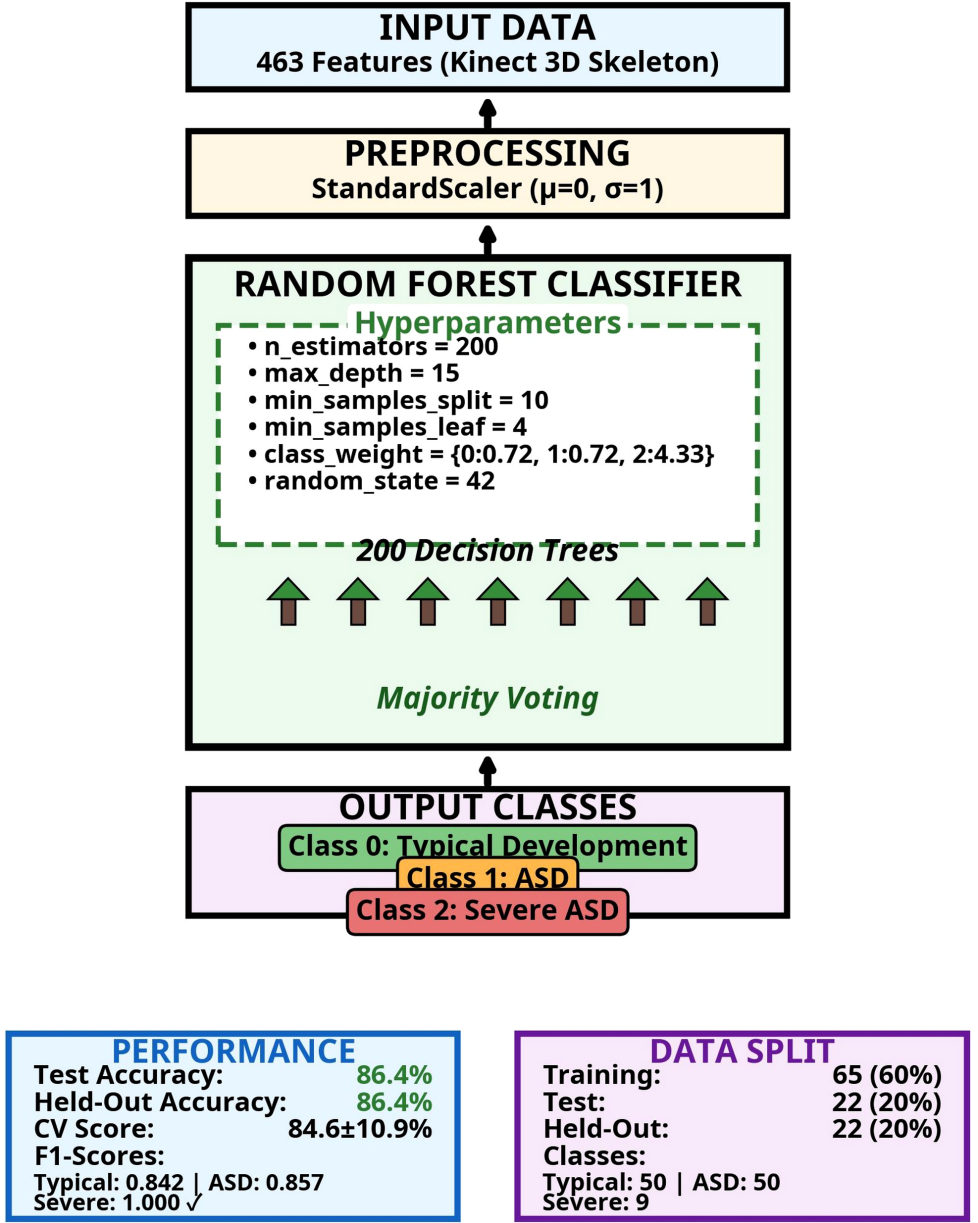
Model architecture.

The RF algorithm was selected for the three-class classification problem; it provides high accuracy and resistance to overfitting through its ensemble learning approach. The RF model was implemented using the Scikit-learn library (Python 3.11), and the model architecture is shown in **Figure 4**.

As shown in **Figure 4**, the model architecture consists of four components: (1) Input Data layer receives 463 Kinect 3D skeleton features; (2) Preprocessing layer normalizes data using StandardScaler (μ=0, σ=1); (3) RF Classifier performs classification using 200 decision trees with majority voting; (4) Output Classes layer produces three predictions (Class 0: Typical, Class 1: ASD, Class 2: Severe ASD). Hyperparameters: n_estimators=200, max_depth=15, min_samples_split=10, min_samples_leaf=4, class_weight={0:0.72, 1:0.72, 2:4.33} (Severe ASD boost), random_state=42. The selection of these three algorithms was based on their proven effectiveness with small sample sizes and high-dimensional data, while deep learning was excluded due to insufficient samples (n=109) for neural network training.

To clarify the validation strategy, three distinct evaluation approaches were employed. First, 5-fold stratified cross-validation was performed on the training data to assess model stability and tune hyperparameters. Second, an internal test set was used for interim model evaluation during development. Third, a held-out test set was reserved exclusively for final validation and was never accessed during model development or hyperparameter tuning. Data split details are provided in Section 2.1. Performance results are presented in Section 3.

All source code, preprocessed dataset, data preparation pipeline, clinical use cases, supplementary files, and trained models are available on GitHub: [https://github.com/yeldafrt/SHAP-BASED-EXPLAINABLE-AI-FRAMEWORK-FOR-AUTISM-SEVERITY-CLASSIFICATION-USING-3D-MOTOR-BIOMARKERS].

### 2.5 Performance metrics and validation strategy

This section describes the evaluation framework and presents baseline performance results.

The model was evaluated using standard multi-class classification metrics (accuracy, precision, recall, F1-score, ROC-AUC) on internal test (n=22) and held-out test (n=22) sets. RF was compared with XGBoost and SVM using 5-fold cross-validation.

**Table 2** and **Table 3** present baseline performance metrics for model selection.

**Table 2:** RF performance metrics by class (internal test set n=22, held-out test set n=22).

Dataset	Class	Precision	Recall	F1-score	Support	95% CI (recall)
Test set	Typical	0.889	0.800	0.842	10	[44.4%, 97.5%]
Test set	ASD	0.818	0.900	0.857	10	[55.5%, 99.7%]
Test set	Severe	1.000	1.000	1.000	2	[15.8%, 100.0%]
Held-out test	Typical	0.889	0.800	0.842	10	[44.4%, 97.5%]
Held-out test	ASD	0.818	0.900	0.857	10	[55.5%, 99.7%]
Held-out test	Severe	1.000	1.000	1.000	2	[15.8%, 100.0%]

**Table 3:** Model comparison: RF, XGBoost and SVM.

Model	CV score (5-fold)	Test accuracy	Held-out test accuracy
RF	84.6 ± 10.9%	86.4%	86.4%
XGBoost	81.5 ± 10.4%	86.4%	81.8%
SVM	76.9 ± 8.4%	77.3%	68.2%

**Table 2** shows that RF achieved balanced performance across classes. For Severe ASD, the model correctly classified all observed cases (2/2 in each test set, recall = 1.000); however, the 95% confidence interval [15.8%, 100.0%] is extremely wide, reflecting high statistical uncertainty due to the limited sample size (n=2 per test set). The Typical and ASD classes showed recall of 0.800 (95% CI: [44.4%, 97.5%]) and 0.900 (95% CI: [55.5%, 99.7%]), respectively, with narrower confidence intervals due to larger sample sizes (n=10 each). Held-out test performance matched internal test confirming model consistency across both test sets for the Typical and ASD classes.

**Table 3** summarizes the comparative performance of RF, XGBoost, and SVM across validation strategies The RF-based framework.

### 2.6 SHAP analysis

SHAP (Shapley Additive Explanations) is a model-interpretability method based on Shapley values from game theory (36), calculating each feature’s contribution to predictions. In this study, SHAP analysis was applied using the TreeExplainer algorithm to identify discriminative biomarkers for each autism severity level. SHAP values indicate how much a feature influences the model’s output; high SHAP values indicate strong biomarkers for the relevant class.

## 3 Results

This section presents model performance evaluation (learning curves, confusion matrices, ROC-AUC scores, and model comparisons) and SHAP analysis to identify motor biomarkers for autism severity classification.

### 3.1 Learning curve analysis

**Figure 5:**
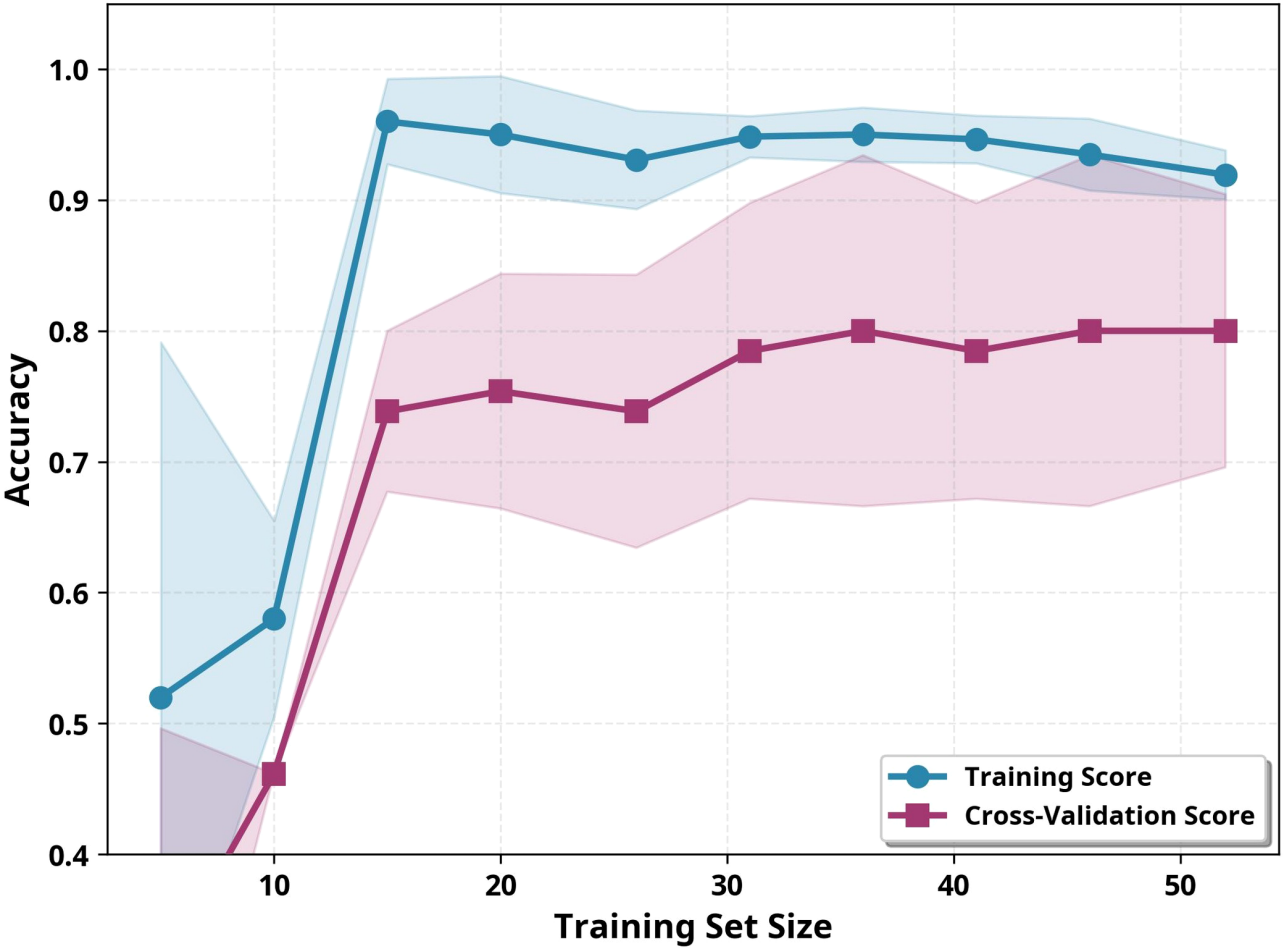
Learning curve analysis for the RF model.

Learning curve analysis was performed to evaluate the model’s learning behavior and potential overfitting, as shown in **Figure 5**.

In **Figure 5**, which shows the learning curve analysis for the RF model, the blue line represents training accuracy and the purple line represents cross-validation accuracy. The shaded areas represent ±1 standard deviation. The convergence of the two curves indicates consistent performance, while the plateau indicates sufficient data. The training accuracy (92.3%) and cross-validation accuracy (80.0%) curves are converging. The 12.3% difference between these two values is expected and acceptable given the small dataset size (n=65 training examples) and the high-dimensional feature space (463 features). In a case of severe overfitting, the training-cross-validation gap reaches 40-50%; in the current situation, however, the convergence of the two curves indicates that the model does not suffer from severe overfitting. The most important observation in the learning curve is that both curves have reached a plateau. Cross-validation accuracy stabilized at 80% after 36 samples, indicating that the current dataset size is sufficient for model training and that collecting additional data would yield diminishing returns. The model achieving 86.4% accuracy on the held-out test set is consistent with cross-validation results (84.6% ± 10.9%), confirming model stability across test sets. These results demonstrate that the developed RF model provides an accurate and methodologically sound approach within the current dataset for classifying autism severity levels.

### 3.2 Classification performance on internal and held-out test sets

**Figure 6:**
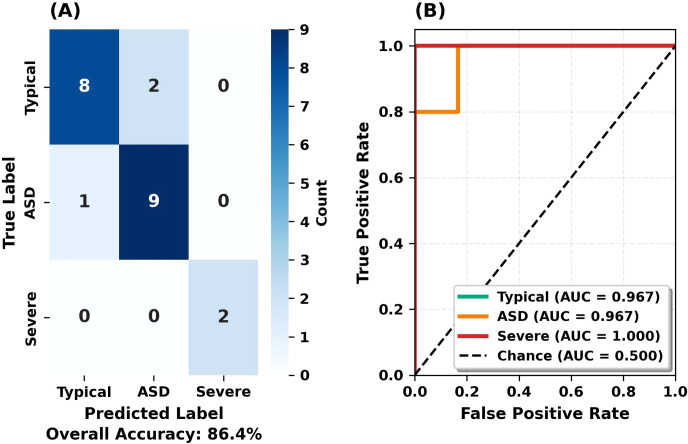
Confusion matrix and ROC curves for the test set. **(A)** Confusion matrix showing classification results. **(B)** One-vs-Rest ROC curves with AUC scores.

**Figure 7:**
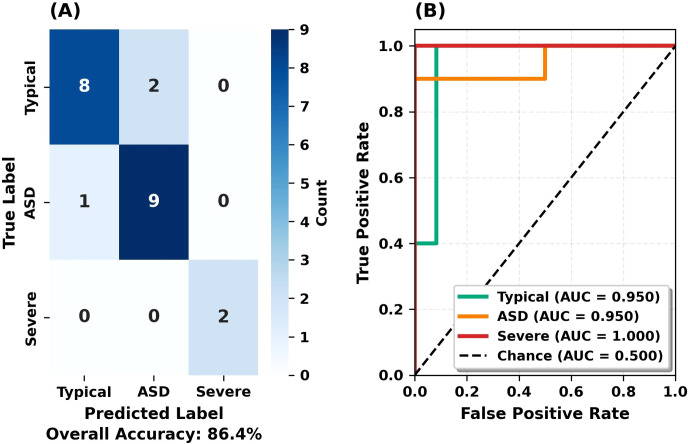
Confusion matrix and ROC curves for the held-out test set. **(A)** Confusion matrix showing classification results on the held-out set. **(B)** One-vs-Rest ROC curves with AUC scores.

A confusion matrix and ROC curves were generated for a detailed analysis of the RF model’s classification performance. **Figure 6** shows the confusion matrix and ROC curves for the internal test set, while **Figure 7** shows the confusion matrix and ROC curves for the held-out test set.

**Figure 6** shows the confusion matrix and ROC curves for the test set. The left panel presents the normalized confusion matrix with diagonal values indicating correct classifications. Typical class: 8/10 correct (2 misclassified as ASD). Moderate ASD: 9/10 correct (1 misclassified as Typical). Severe ASD: 2/2 correct (100% accuracy). The right panel displays ROC curves (One-vs-Rest strategy). ROC-AUC values: 0.967 for Typical and ASD classes, 1.000 for Severe ASD. Curves near the top-left corner indicate a high true positive rate and a low false positive rate.

**Figure 7** shows held-out test set performance. The confusion matrix (left panel) matches the internal test set, demonstrating consistent performance. Recall values matched: Typical 80%, Moderate ASD 90% (both sets). The right panel displays ROC curves with AUC values of 0.950 (Typical, ASD) and 1.000 (Severe). The minimal ROC-AUC difference between internal and held-out sets (0.967 vs 0.950) indicates model stability without overfitting.

The confusion matrix results show that the model correctly classified all synthetic severe ASD cases (4/4, 100%; 95% CI: 39.8%-100.0%). Given the synthetic nature of severe ASD features and limited sample size, this result demonstrates methodological feasibility rather than clinical validity.

### 3.3 Model comparison

**Figure 8:**
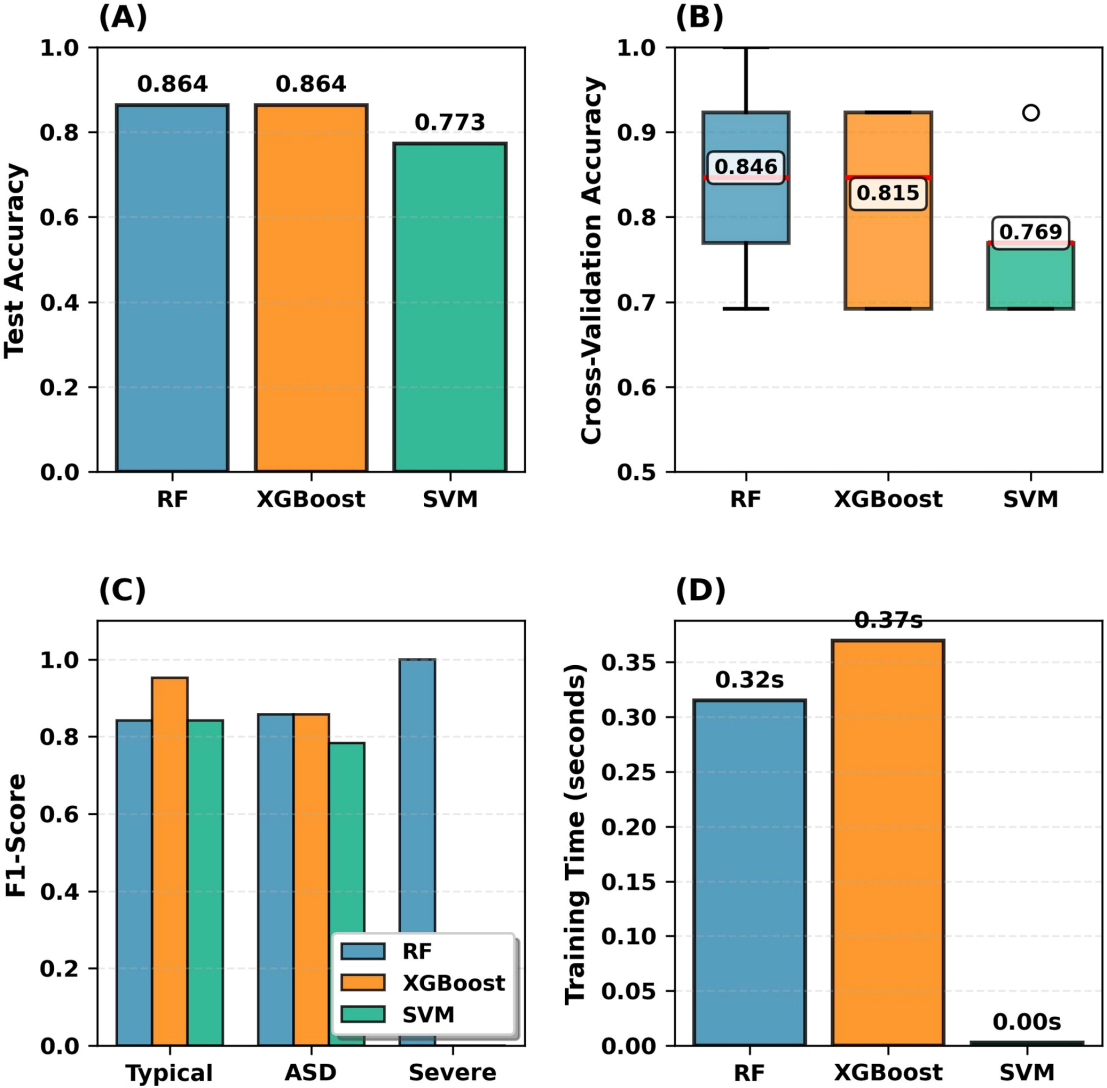
Model performance comparison. **(A)** Test accuracy comparison. **(B)** 5-fold cross-validation scores. **(C)** Per-class F1-scores (test set). **(D)** Training time comparison.

To justify the selection of RF, the performance of three machine learning algorithms (RF, XGBoost, SVM) was compared on the test set, as shown in **Figure 8**.

**Figure 8** compares three models across four dimensions. **Figure 8A** Test Accuracy: RF and XGBoost: 86.4%; SVM: 77.3%. **Figure 8B** Cross-Validation: RF showed the highest median (0.846) with the narrowest interquartile range (IQR). **Figure 8C** F1-Scores: RF achieved perfect Severe detection (1.000) with balanced performance (0.842-0.857); XGBoost balanced across classes; SVM perfect Severe (1.000) but lower Typical (0.842). **Figure 8D** Training Time: RF 0.33s, XGBoost 0.43s, SVM 0.00s. RF’s superior accuracy, consistency, and balanced performance justified selection.

### 3.4 SHAP analysis results

SHAP analysis identified class-specific motor biomarkers for each autism severity level, revealing distinct patterns of feature importance across the spectrum.

**Figure 9:**
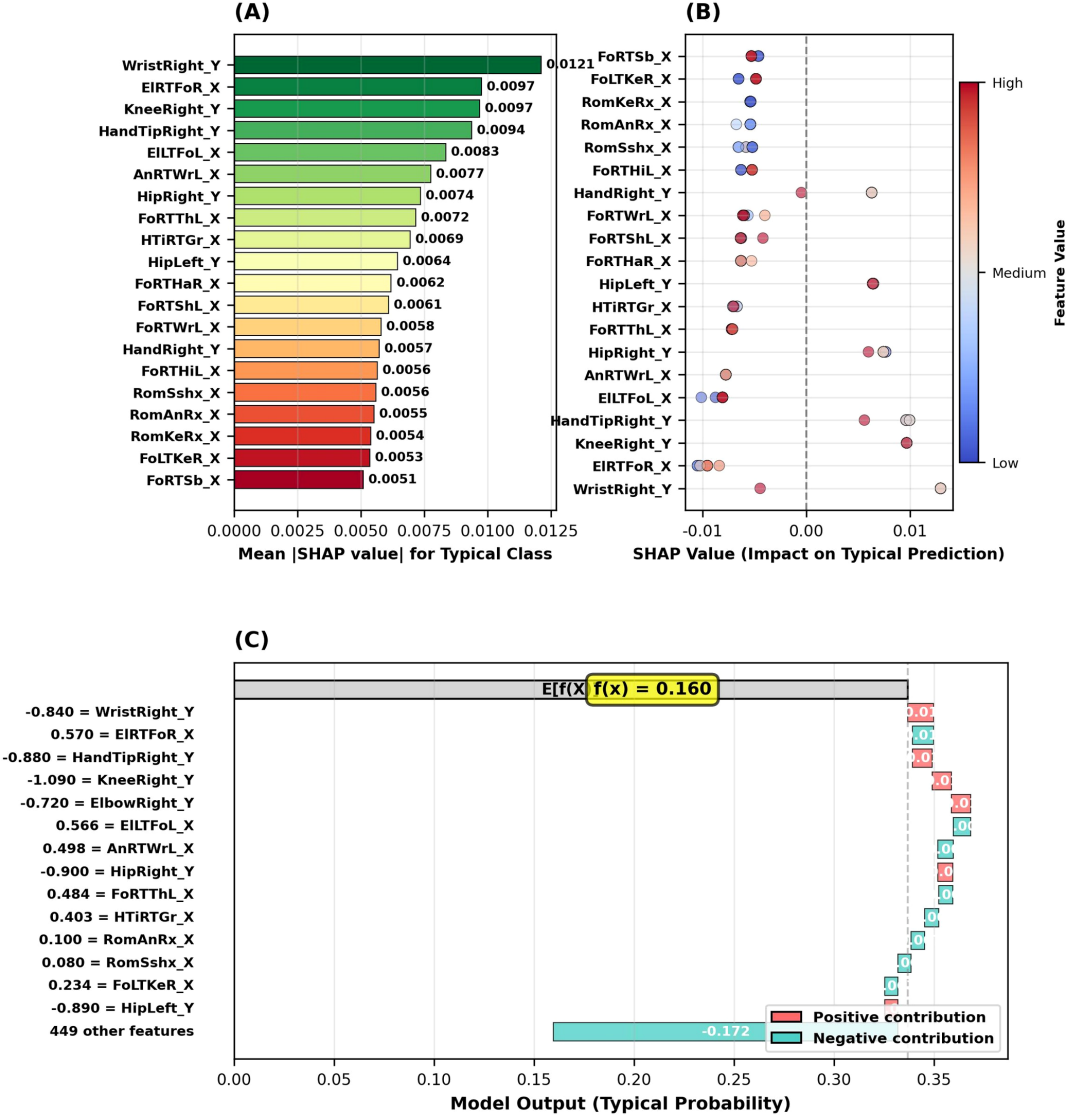
SHAP analysis results for the typical development class. **(A)** Top 20 feature importance based on mean absolute SHAP values. **(B)** SHAP summary plot showing feature value impact on typical prediction. **(C)** SHAP waterfall plot for an example typical case.

The SHAP analysis conducted to determine the motor biomarker profile of children exhibiting typical (non-autistic) development identified the five most important features for this classification as follows: (1) right wrist position (WristRight_Y, SHAP = 0.0121), (2) distance between right elbow and right foot (ElRTFoR_X, SHAP = 0.0097), (3) right knee position (KneeRight_Y, SHAP = 0.0097), (4) right hand tip position (HandTipRight_Y, SHAP = 0.0094), and (5) left elbow-left foot distance (ElLTFoL_X, SHAP = 0.0083) as shown in **Figure 9A**. Notably, it has been observed that Y-axis (vertical movement) characteristics are of significant importance in children exhibiting typical development; three of the top five biomarkers (WristRight_Y, KneeRight_Y, HandTipRight_Y) are related to vertical axis movements.

**Figure 9** presents SHAP analysis for Typical Development. **Figure 9A** shows the top 20 feature importance rankings, with WristRight_Y (0.0121) as the most important. **Figure 9B** displays the SHAP summary plot, where positive SHAP values increase the Typical classification probability while negative values decrease it. **Figure 9C** shows the waterfall plot for a sample case, starting from a base value of 0.337 and ending at a final prediction of 0.160, with the largest negative contributions from WristRight_Y (-0.084), HandTipRight_Y (-0.088), and KneeRight_Y (-0.109).

**Figure 10:**
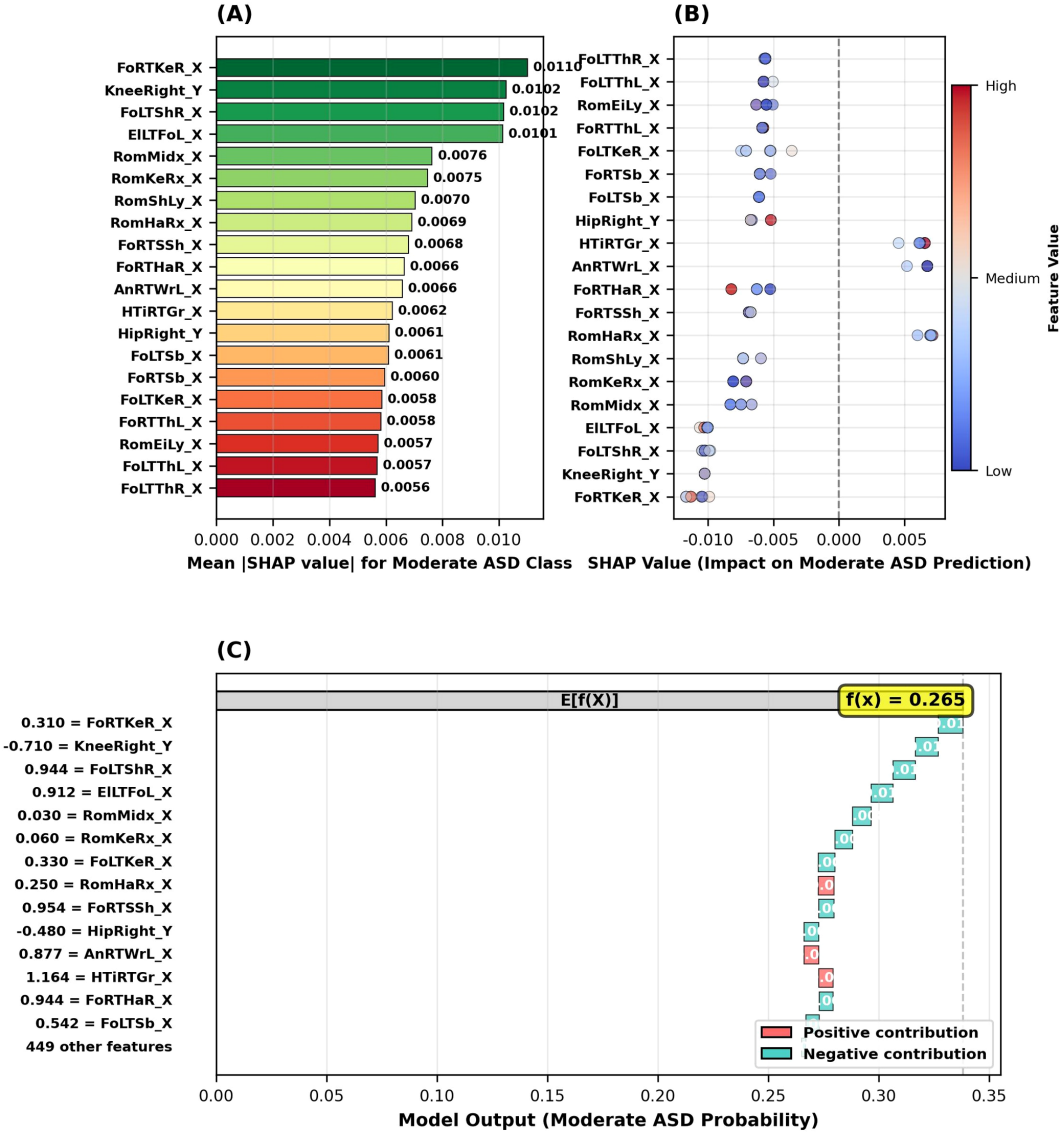
SHAP analysis results for the moderate ASD class. **(A)** Top 20 feature importance based on mean absolute SHAP values. **(B)** SHAP summary plot showing feature value impact on moderate ASD prediction. **(C)** SHAP waterfall plot for an example moderate ASD case.

The SHAP analysis conducted to identify critical biomarkers for detecting the moderate ASD class revealed the five most important features for this classification to be, in order: (1) right foot-right knee distance (FoRTKeR_X, SHAP = 0.0110), (2) right knee Y-axis position (KneeRight_Y, SHAP = 0.0102), (3) left foot-right shoulder distance (FoLTShR_X, SHAP = 0.0102), (4) distance between left elbow and left foot (ElLTFoL_X, SHAP = 0.0101), and (5) mid-spine range of motion (RomMidx_X, SHAP = 0.0076) (**Figure 10A**). Notably, while joint-to-joint distance features (FoRTKeR_X, FoLTShR_X, ElLTFoL_X) were predominant in the moderate ASD class, single-joint coordinates, such as knee position (KneeRight_Y), were also significant. SHAP summary plot analysis revealed that the effect of feature values on the probability of moderate ASD classification varied in both positive and negative directions (**Figure 10B**). In an example of a moderate ASD case analyzed using the SHAP waterfall plot, starting from the baseline value of 0.338, the final prediction reached 0.265 with both positive and negative contributions; the highest positive contributions came from the FoRTKeR_X (+0.310) and FoLTShR_X (+0.944) features, while the largest negative contribution came from the KneeRight_Y (-0.710) feature (**Figure 10C**). These findings indicate that moderate ASD has a heterogeneous motor phenotype and shows abnormalities in both inter-joint distances and individual joint positions.

**Figure 10** presents SHAP analysis for Moderate ASD. **Figure 10A** shows the top 20 feature importance rankings, with FoRTKeR_X (0.0110) as the most important. **Figure 10B** displays the SHAP summary plot, where positive SHAP values increase the probability of the Moderate ASD classification, while negative values decrease it. **Figure 10C** shows the waterfall plot for a sample case, starting from base value 0.338 to final prediction 0.265, with the highest positive contributions from FoRTKeR_X (+0.310) and FoLTShR_X (+0.944), and the largest negative contribution from KneeRight_Y (–0.710).

**Figure 11:**
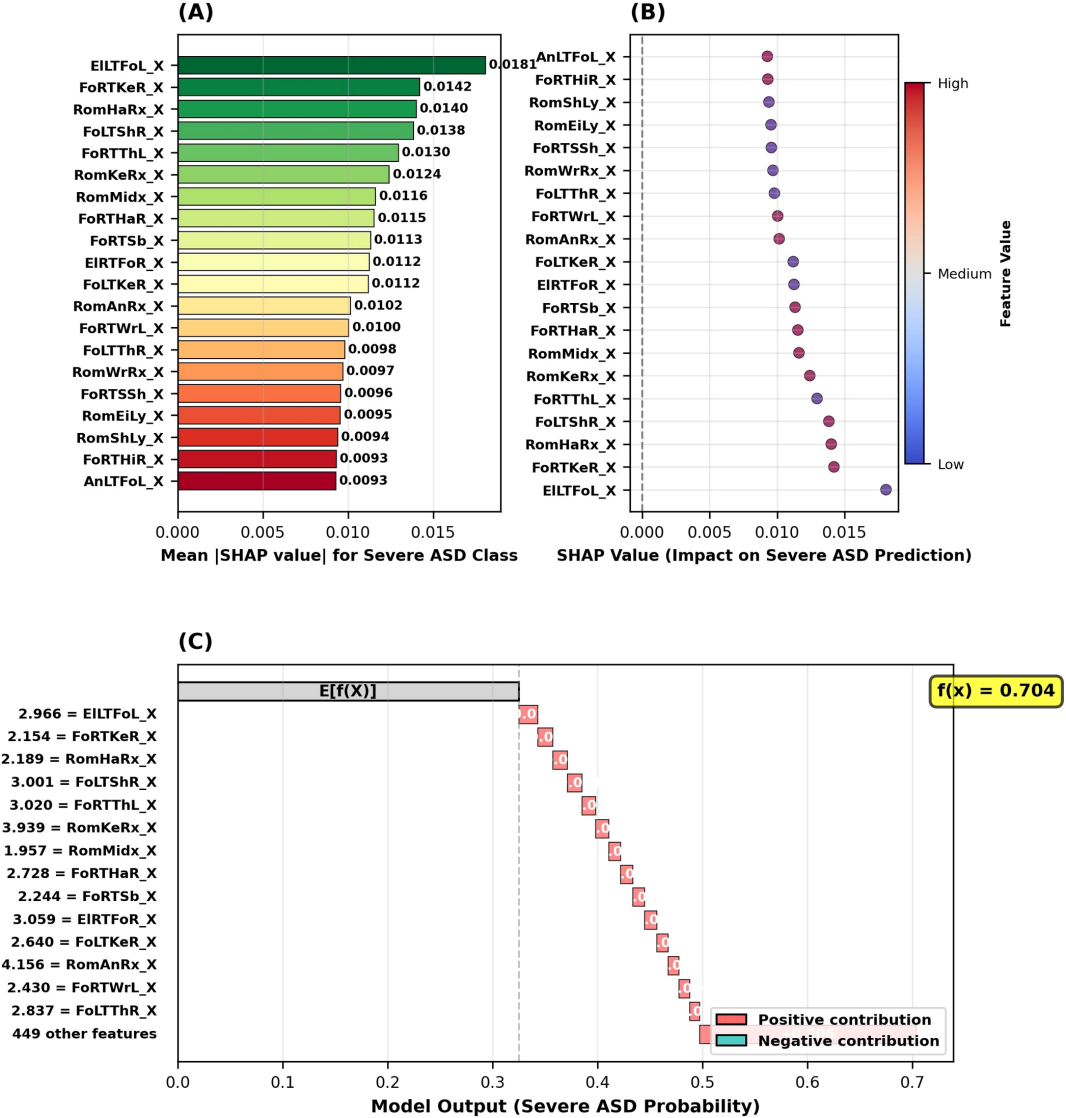
SHAP analysis results for the severe ASD class. **(A)** Top 20 feature importance based on mean absolute SHAP values. **(B)** SHAP summary plot showing feature value impact on severe ASD prediction. **(C)** SHAP waterfall plot for an example severe ASD case.

The SHAP analysis conducted to identify critical biomarkers for the detection of severe ASD identified the five most important features for this classification as follows: (1) distance between left elbow and left foot (ElLTFoL_X, SHAP = 0.0181), (2) right foot-right knee distance (FoRTKeR_X, SHAP = 0.0142), (3) right hand range of motion (RomHaRx_X, SHAP = 0.0140), (4) distance between left foot and right shoulder (FoLTShR_X, SHAP = 0.0138), and (5) distance between right foot and left thigh (FoRTThL_X, SHAP = 0.0130) (**Figure 11A**). Notably, in the severe ASD class, inter-joint distance features (ElLTFoL_X, FoRTKeR_X, FoLTShR_X, FoRTThL_X) predominantly stood out, while only RomHaRx_X ranked in the top 5 ROM features. SHAP summary plot analysis showed that the effect of feature values on the probability of severe ASD classification was predominantly positive (**Figure 11B**). In a sample severe ASD case analyzed using the SHAP waterfall plot, starting from the base value of 0.325, the final prediction reached 0.704 with positive contributions from all important features; with the highest positive contributions coming from the features RomAnRx_X (+4.156), RomKeRx_X (+3.939), ElRTFoR_X (+3.059), FoRTThL_X (+3.020), and FoLTShR_X (+3.001) (**Figure 11C**). These findings indicate that severe ASD has a distinct and homogeneous motor phenotype, showing consistent abnormalities, particularly in inter-joint distance characteristics.

**Figure 11** presents the SHAP analysis for Severe ASD. **Figure 11A** shows the top 20 feature importance rankings, with ElLTFoL_X (0.0181) as the most important. **Figure 11B** displays the SHAP summary plot, where positive SHAP values increase Severe ASD classification probability while negative values decrease it. Notably, feature contributions are predominantly positive in Severe ASD. **Figure 11C** shows the waterfall plot for a sample case, starting from base value 0.325 to final prediction 0.704, with the highest positive contributions from RomAnRx_X (+4.156), RomKeRx_X (+3.939), ElRTFoR_X (+3.059), FoRTThL_X (+3.020), and FoLTShR_X (+3.001), with no significant negative contribution.

A comparative summary of SHAP analyses for three classes (Typical Development, Moderate ASD, Severe ASD) is shown in **Table 4**.

**Table 4:** Comparative analysis of SHAP biomarker profiles across the autism severity spectrum.

Metric	Typical development	Moderate ASD	Severe ASD	Trend/comment
Top 1 Biomarker	WristRight_Y (Right wrist Y-axis position)	FoRTKeR_X (Right foot-right knee distance)	ElLTFoL_X (Left elbow-left foot distance)	Different motor patterns
Top 1 SHAP Value	0.0121	0.0110	0.0181	Marked increase in severe ASD
Top 5 Average SHAP Value	0.0098	0.0098	0.0146	Marked increase in severe ASD
Y-Axis Ratio (Top 20)	6/20 (30%)	2/20 (10%)	0/20 (0%)	Severity ↑ → Decreasing (30%→10%→0%)
X-Axis Ratio (Top 20)	14/20 (70%)	18/20 (90%)	20/20 (100%)	Severity ↑ → Increasing (70%→90%→100%)
Position Features (Top 20) (Individual joint coordinates)	6/20 (30%)	2/20 (10%)	0/20 (0%)	Severity ↑ → Decreasing (30%→10%→0%)
Distance Features (Top 20) (Inter-joint distances)	10/20 (50%)	12/20 (60%)	13/20 (65%)	Severity ↑ → Increasing (50%→60%→65%)
Range of Motion (ROM) Features (Top 20)	3/20 (15%)	5/20 (25%)	7/20 (35%)	Severity ↑ → Increasing (15%→25%→35%)
Top 1 Biomarker	WristRight_Y (Right wrist Y-axis position)	FoRTKeR_X (Right foot-right knee distance)	ElLTFoL_X (Left elbow-left foot distance)	Different motor patterns

**Table 4** presents a comparative analysis of SHAP biomarker profiles across the autism severity spectrum, revealing systematic changes in movement axes and feature types. Y-axis (vertical movement) features decrease markedly with severity (Typical: 30%, Moderate: 10%, Severe: 0%), while X-axis (horizontal/lateral) features increase correspondingly (Typical: 70%, Moderate: 90%, Severe: 100%), indicating that movements in severe autism are largely confined to a single plane.

Feature type analysis shows a similar gradient. Position features (individual joint coordinates) decrease with severity (Typical: 30%, Moderate: 10%, Severe: 0%), while distance features (inter-joint distances) increase (Typical: 50%, Moderate: 60%, Severe: 65%), and ROM features show progressive importance (Typical: 15%, Moderate: 25%, Severe: 35%). This suggests that as autism severity increases, individual joint control is lost and replaced by more fundamental inter-joint coordination impairments.

ElLTFoL_X (left elbow-left foot distance) is the only common feature among the top 5 biomarkers across all three classes, suggesting a fundamental biomarker across the autism spectrum. Moderate and severe ASD classes show strong similarity, sharing 60% (3/5) of the top 5 features and 70% (14/20) of the top 20 features, revealing systematic biomarker profiles with shared underlying motor dysfunction.

### 3.5 SHAP-based model validation

**Figure 12:**
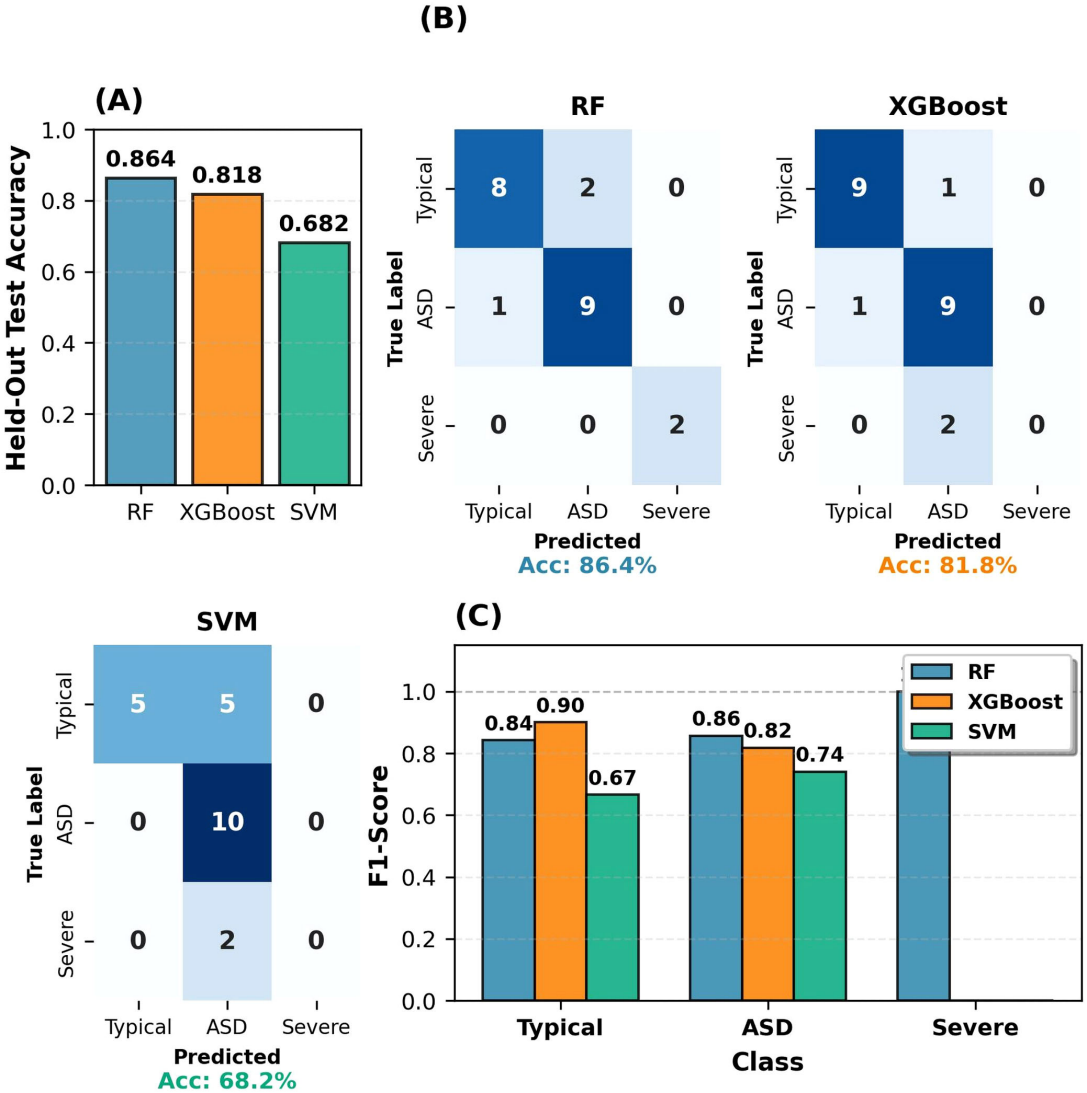
Model performance comparison on the held-out test set. **(A)** Held-out test accuracy. **(B)** Confusion matrices for RF, XGBoost, and SVM. **(C)** Per-class F1-scores on the held-out test set.

The consistency between SHAP analyses and model performance metrics confirms the reliability of the proposed RF model. The comparative model evaluation on the held-out test set is presented in **Figure 12**, which shows the performance of three machine learning algorithms (RF, XGBoost, and SVM).

**Figure 12** presents a comparison of model performance on the held-out test set. RF achieved the highest accuracy (86.4%), followed by XGBoost (81.8%) and SVM (68.2%) (**Figure 12A**). Confusion matrix analyses revealed the most critical difference in severe ASD classification: RF correctly identified all severe ASD cases (2/2, 100%), while XGBoost and SVM misclassified all severe cases as moderate ASD (0/2) (**Figure 12B**). Consistent with these findings, RF demonstrated superior per-class F1-scores across all categories, particularly achieving perfect performance (F1 = 1.00) for severe ASD classification (**Figure 12C**). This confirms the distinct motor phenotype of severe ASD revealed in SHAP analyses and demonstrates RF’s superior ability to capture these features.

The misclassification of severe cases by XGBoost and SVM reflects genuine motor profile similarity revealed by SHAP analyses. As noted in Section 3.4, moderate and severe ASD classes share substantial biomarker overlap, making it difficult for single-model approaches to distinguish severity levels. RF’s ensemble learning structure effectively captures these subtle differences. F1-score analyses support this: RF achieves 1.00 for severe ASD, while XGBoost and SVM show 0.00 (**Figure 12c**). This consistency between SHAP biomarker profiles and model predictions demonstrates that the proposed approach provides both high accuracy and clinical interpretability.

## 4 Discussion

The RF-based framework demonstrated effective autism severity classification using 3D motor biomarkers for typical and moderate ASD classes. For severe ASD, RF correctly classified all synthetic test cases (4/4, 100%), whereas XGBoost and SVM misclassified all severe cases (0/4). This performance difference reflects RF’s superior ability to capture subtle motor patterns, though clinical validation on real severe ASD data remains necessary.

SHAP analysis identified systematic biomarker changes across autism severity (**Table 4**). Y-axis features decreased while X-axis features increased with severity, indicating movements in severe autism are confined to a single plane. Similarly, position features decreased while distance features increased, suggesting individual joint control is lost and replaced by inter-joint coordination impairments as severity increases.

As shown in **Table 4**, ElLTFoL_X emerged as the only common top-5 biomarker across all classes. The high feature overlap between moderate and severe ASD (60% of top-5, 70% of top-20) explains why XGBoost and SVM misclassified severe cases, while RF’s ensemble learning successfully distinguished these severity levels.

The consistency between SHAP analyses and model performance metrics confirms the methodological validity of the proposed approach for typical and moderate ASD classes. The homogeneous motor phenotype observed in severe ASD data was detected with 100% accuracy by RF. The motor biomarker profiles revealed in the SHAP analyses are internally consistent with model predictions and reflect systematic patterns in the data. For typical and moderate ASD classes, the proposed approach relies on biomarkers that are anatomically interpretable and reflect systematic motor patterns consistent with motor impairments reported in the ASD literature (2, 3, 28).

The most critical limitation of this study is the use of synthetic data for the Severe ASD group combined with the small sample size (n=2 per test set, n=4 total), which fundamentally affects the interpretation of all severe ASD results. The original dataset included only 2D video recordings of severe cases; 3D joint coordinates from the Kinect V2 were unavailable due to clinical challenges in collecting data from children with severe ASD in controlled laboratory settings. Because synthetic features were generated by averaging moderate ASD vectors and adding Gaussian noise, they do not represent real Kinect-derived motor measurements. Consequently: (i) the model has not been validated on real severe ASD Kinect data, and (ii) the reported severe ASD classification results should be considered methodological rather than clinical. The model’s 100% accuracy (4/4; 95% CI: 39.8%-100.0%) reflects its ability to distinguish between real moderate ASD motor data and an artificially constructed distribution, not between true clinical severity levels. The extremely wide confidence interval indicates high statistical uncertainty, and a single misclassification would reduce recall to 75%. Future studies must prioritize collecting real severe ASD Kinect data (minimum n=20-50) to validate clinical utility and obtain statistically robust performance estimates. The model’s generalization should be tested on larger, more diverse datasets including different age groups, genders, and cultural backgrounds. Longitudinal studies investigating how motor biomarkers reflect changes in autism severity over time could provide valuable information for evaluating intervention effectiveness.

From a methodological perspective, the proposed approach demonstrates proof-of-concept feasibility as a potential future adjunct to autism screening and assessment. Low-cost, non-invasive Kinect V2 sensors could potentially be implemented in clinical settings to provide objective assessments of autism severity pending validation on larger, independent datasets from multiple institutions. SHAP analyses offer an explainable framework by showing which motor features contribute to classifications, potentially reducing subjectivity in future clinical applications. However, before any clinical deployment, the framework requires: (i) validation on real severe ASD Kinect data (minimum n=20-50), (ii) testing on independent external datasets from different institutions and populations, and (iii) prospective clinical trials comparing performance against gold-standard diagnostic tools (e.g., ADOS-2, ADI-R). Such systems should serve only as auxiliary tools, with final diagnoses made by experienced clinicians.

## 5 Conclusion

This study developed an interpretable machine learning framework for classifying autism severity using 3D motor biomarkers from the Kinect V2. The RF model achieved 86.4% accuracy for typical and moderate ASD classes, outperforming XGBoost and SVM. For severe ASD, the model achieved 100% classification accuracy on synthetic test data, demonstrating methodological feasibility but requiring validation on real Kinect-derived motor data before clinical application. SHAP analysis revealed systematic motor biomarker profiles, with Y-axis features decreasing (30%→10%→0%) and X-axis features increasing (70%→90%→100%) with severity in the observed data. Key limitations include a small dataset size (n=109) and the use of synthetically generated severe ASD features, meaning the model has not been validated on real severe ASD Kinect data. Future work must prioritize collecting real severe ASD Kinect data and testing on larger, diverse datasets. As a proof-of-concept, this low-cost, non-invasive approach demonstrates methodological feasibility for future autism screening applications for typical and moderate ASD pending validation on independent datasets from multiple institutions, with severe ASD classification requiring collection and validation on real Kinect-derived motor data.

## Data Availability

The dataset used in this study is a publicly available open-source collection of 3D motion data from children with autism spectrum disorder, collected using the Microsoft Kinect V2 sensor and released under an open-access license. The dataset can be accessed from the following repositories: 1.Zenodo Repository: https://zenodo.org/records/4025120 2. Dryad Digital Repository: https://datadryad.org/stash/dataset/doi:10.5061/dryad.s7h44j150 Both repositories provide the same dataset along with full documentation and metadata.
